# P03.14. Cancer complementary and alternative medicine research among NCI’s cancer centers program and the integrative medicine programs: an inventory

**DOI:** 10.1186/1472-6882-12-S1-P267

**Published:** 2012-06-12

**Authors:** I Mikhail, E Austin, S Buckman, C Lee, N Goodman, J White

**Affiliations:** 1 National Cancer Institute-DCTD-Office of Cancer CAM (OCCAM), Bethesda, USA; 2 National Cancer Institute, Office of Cancer Education (OCE), Bethesda, USA

## Purpose

The National Cancer Institute (NCI) supports a Cancer Centers Program (CCP) including 66 NCI-designated cancer centers. Several CCPs have associations with Integrative Medicine Programs (IMPs). However, limited information is available regarding cancer-CAM research and collaborations between them. An inventory by NCI’s Office of Cancer Complementary and Alternative Medicine (OCCAM) was conducted to learn about CAM related services and resources at NCI’s CCPs and IMPs, including members of the Consortium of Academic Health Centers for Integrative Medicine (CAHCIM).

## Methods

Sixty-six NCI-designated cancer centers of the CCP and 41 IMPs were eligible to participate in a brief inventory about clinical services, education, and research. This project examines the CCP-IMP cancer research results of this inventory.

## Results

Most CCPs and IMPs indicated collaborating with each other and also working independently. CCPs conducted more independent research (38%) than IMPs (28%). Types of research collaborations included mainly clinical research (93% at CCPs; 88% at IMPs). International collaborations were higher among CCPs (27%) compared to IMPs (11%). Both CCPs and IMPs reported NIH as their major source of funding (76% and 59%, respectively). About 70% of CCPs and IMPs reported having CAM research experts in their centers. When asked if their institutions would find an NCI training on cancer CAM research grants beneficial, both CCP (85%) and IMP (94%) respondents were interested in such training and considered it to be beneficial.

## Conclusion

This inventory shows that research collaborations between CCPs and IMPs are ongoing. However, additional in depth details are needed. Research training on cancer CAM grants can be beneficial in fostering research collaborations and in advancing cancer CAM research among CCPs and IMPs.

